# Lower dietary mineral intake is significantly associated with cervical cancer risk in a population-based cross-sectional study

**DOI:** 10.7150/jca.39806

**Published:** 2021-01-01

**Authors:** Zhe Wang, Wenhao Wang, Aimin Yang, Weihong Zhao, Jing Yang, Zhilian Wang, Wei Wang, Xiaoqiang Su, Jintao Wang, Jinghui Song, Li Li, Weiguo Lv, Dongyan Li, Huiqiang Liu, Chen Wang, Min Hao

**Affiliations:** 1 Department of Obstetrics and Gynecology, Second Hospital of Shanxi Medical University, Taiyuan, Shanxi, China.; 2 Hong Kong Institute of Diabetes and Obesity, The Chinese University of Hong Kong, Hong Kong SAR.; 3 Department of Epidemiology, School of Public Health, Shanxi Medical University, Taiyuan, China.; 4 Department of Obstetrics and Gynecology, Affiliated Hospital of Inner Mongolia Medical University, Huhhot, China.; 5 Department of Obstetrics and Gynecology, Affiliated Tumor Hospital of Guangxi Medical University, Nanning, China.; 6 Department of Gynecologic Oncology, Women's Hospital, School of Medicine, Zhejiang University, Hangzhou, Zhejiang, China.; 7 Department of pathology, Second Hospital of Shanxi Medical University, Taiyuan, Shanxi 030001, China.

**Keywords:** dietary mineral intake, cervical intraepithelial neoplasia, cervical cancer, China, cross-sectional study

## Abstract

Population-based studies investigating the association between dietary mineral intake and risk of cervical intraepithelial neoplasia (CIN) or cervical cancer in Chinese women are few. We performed a cross-sectional analysis of screening data obtained from 2,304 women in 2014 within an ongoing cohort study comprising 40,000 women in China. Dietary intake was assessed using a semiquantitative food frequency questionnaire. Nutrition intake was calculated using a 26-item list of food sources drawn from a validated, comprehensive database. All participants were surveyed through in-person interviews, physical examinations, and laboratory tests. The Pearson chi-square test was used for categorical variables. Multivariable logistic regression models were used to evaluate the relationship between dietary mineral intake and CIN+ risk. The food frequency questionnaire exhibited acceptable reproducibility and reasonable validity in assessing nutrient intakes among these women. After adjusting for multiple potential confounders, low dietary calcium intake was associated with CIN2+ risk (first versus fourth quartile: odds ratio [OR]=1.52, 95% confidence interval [CI]: 1.01-2.32). Similar for magnesium (OR=1.80, 95% CI: 1.20-2.68), phosphorus (OR=1.69, 95% CI: 1.12-2.55), zinc (OR=1.55, 95% CI: 1.03-2.34), and potassium (OR=1.92, 95% CI: 1.28-2.88). Low dietary intakes of calcium and potassium were significantly associated with CIN1 risk. Increased CIN2+ risk correlated with rates of no oral contraceptives and lower levels of dietary Potassium. These results thus proposed that low dietary mineral intake was an independent risk factor, potential synergy may exist between low dietary mineral levels and oral contraceptives contribute to the development of higher-grade CIN and cervical cancer.

## Introduction

Cervical intraepithelial neoplasia (CIN) is a precancerous lesion of the cervix^1^ and reflects the initial stage of cervical carcinogenesis^2^. Although advances such as the human papilloma virus (HPV) vaccine and ThinPrep cytologic test have helped to reduce incidence associated with this disease^3^, these procedures are expensive. Hence, cervical cancer remains the most common malignancy among women in low-resource countries^4^. For that reason, the geographical factors influencing cervical cancer are worth investigating in order to improve prevention methods.

HPV is a primary causative agent for cervical cancer^5^, and HPV-associated disease has an incidence rate of 6.6 per 100,000 population worldwide^6^. Recent studies revealed that the risk factors for cervical cancer include not only HPV infection^7^, but also maintaining unhealthy long-term lifestyles, dietary habits, and vaginal microenvironments^8^]-9. However, whether such factors differ by region has rarely been investigated. Diet, which is a region-associated factor and includes parameters such as mineral intake, has recently been reported to be associated with the risk of CIN^10^-^11^. The intake of magnesium, calcium, and zinc is essential for maintaining the integrity of DNA and preventing oxidative DNA damage^12^-^14^; as such, intake deficiency of these minerals can trigger cancer development.

A previous epidemiologic study showed no association between dietary calcium and the risk of cervical cancer^15^. Subsequently, another study found that increased dietary calcium reduced the risk of cervical cancer^5^, because it is protective against HPV infection^16^. A separate study found no association between dietary zinc and the risk of cervical disease^17^, although a subsequent investigation found that dietary zinc reduced the risk of CIN^18^, while low intake of zinc was a risk factor for cervical cancer^19^. Furthermore, dietary phosphorus was found to be significantly associated with cancer development^20^. Although there is no reported direct association between dietary phosphorus and cervical cancer^18^, a significant association was found between the dietary calcium-to-phosphorus ratio and the risk of CIN^15^. Another study found that dietary iron protects against cervical cancer^21^, while other studies found that dietary magnesium and potassium were associated with a reduced risk of other cancers^22^**^־^**^23^. However, the association between dietary magnesium or potassium and the risk of cervical cancer has not been investigated.

Shanxi province in China has a particularly high cervical cancer rate; 10-fold higher than the national average in China^24^. Since it is a landlocked and low income region, its residents often consume unhealthy diets and tend to be malnourished, with poor nutritional intake^25^. However, whether dietary mineral intake influences the incidence of cervical cancer remains controversial^26^. To investigate this, we performed a cross-sectional analysis of screening data obtained in 2014 from 2,304 women derived from an ongoing cohort of 40,000 women in Shanxi province (the Shanxi CIN Cohort). Specifically, we evaluated the association between intake of iron, calcium, magnesium, phosphorus, zinc, sodium, and potassium and CIN risk using categorical analyses.

## Materials and Methods

### Study population

The study data were obtained from the cervical cancer screening program records of 40,000 eligible local women in Shanxi Province, which constituted the baseline survey of the Shanxi CIN Cohort Study. Study conducted free cervical cancer screenings for eligible women permanently residing in two counties of Shanxi province between June 2014 and December 2014^27^. The rationale, design, and methods of the Shanxi CIN Cohort Study have been detailed elsewhere^25^, ^28^.

**Figure 1:**
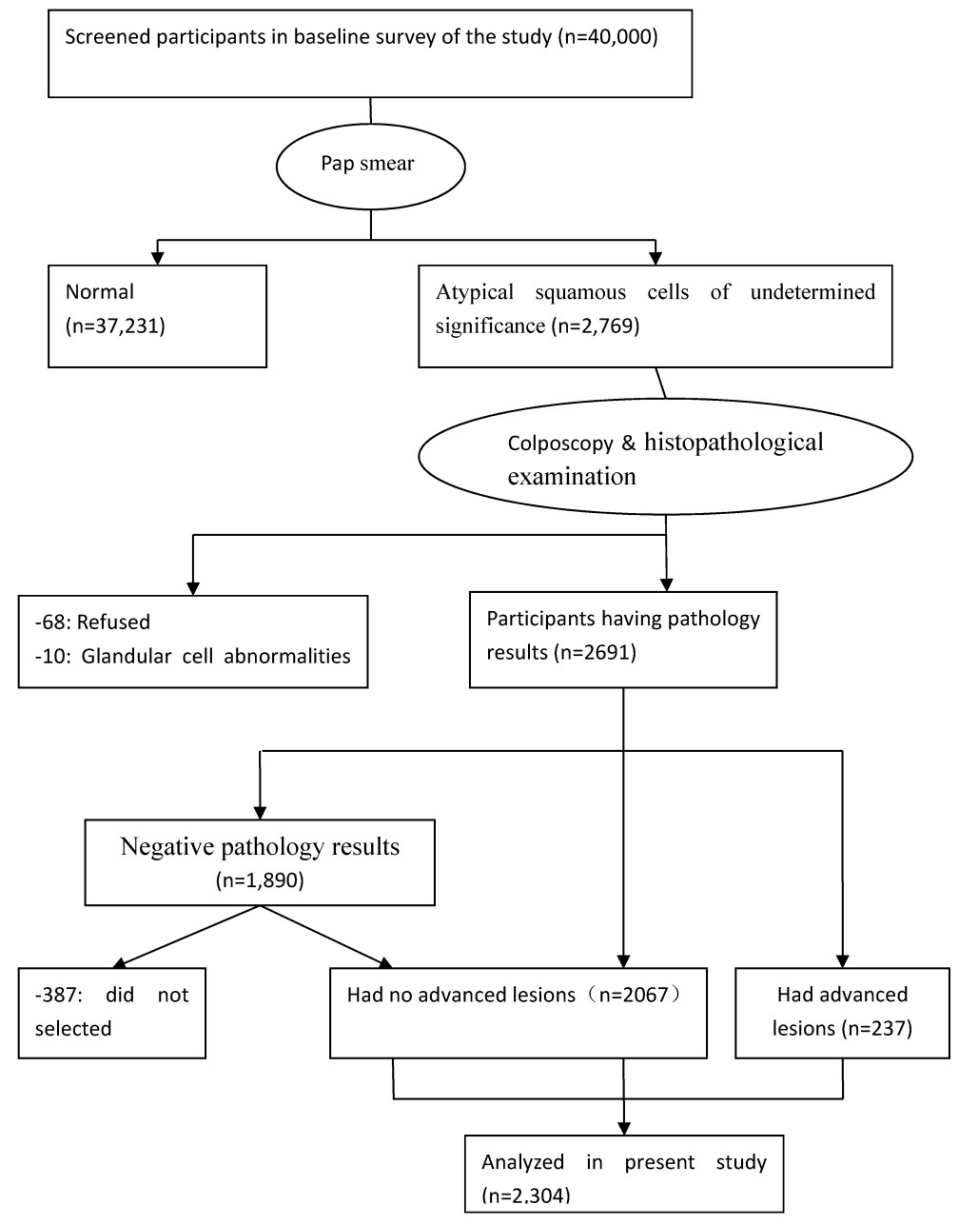
Flow diagram of the participants in the study. ASC-US, atypical squamous cells of undetermined significance; CIN, cervical intraepithelial neoplasia.

Participants were surveyed using a questionnaire of demographic characteristics combined with liquid-based cytology (LBC); colposcopists and histopathologists participated in screening the participants and investigating abnormal results. Of the 40,000 women, 2,769 were diagnosed with atypical squamous cells of undetermined significance and higher-stage lesions, while 78 were excluded (68 refused to continue examination and 10 had glandular cell abnormalities). A total of 2,691 participants underwent colposcopy and histopathological examination; 1,890 women were found to have negative pathology results that with abnormal cytology with currently normal histology. Of the remaining 801 women, 564 has histologically confirmed CIN grade 1 (CIN1) and 237 had CIN grade 2 or higher (CIN2+). Among the 1,890 women with negative pathologic results, 387 were excluded because they had not fully completed the three-part medical examination (in-person interview, physical examination, and clinical examination). Ultimately, 1,503 CIN-negative participants were included. The final sample population comprised 2,304 women (Figure 1). All examinations were performed under double-blind conditions. The study was approved by the ethics committee of the Second Hospital, Shanxi Medical University, and written informed consent was obtained from all the participants. The study was registered in the Chinese Clinical Trial Register (ChiCTR), ChiCTR-ROC-15006479.

### Data collection

The questionnaire-based data in this study were obtained via in-person interviews, while clinical data were obtained via physical examinations, laboratory tests, and the collection of biospecimens. Collected demographic information included age, years of education, annual income, tobacco smoking, age at menarche, menopausal status, years of intrauterine device (IUD) use, sexual activity during the menstrual period, oral contraceptive use, and bathing after sexual intercourse. A food frequency questionnaire (FFQ), which collects information about the frequency and quantity of ingestion of 26 food items, was completed by the participants. Clinical data from physical examinations were used for determining IUD use, squamous-columnar junction (SCJ) visibility, gynecologic surgery, and vaginitis. Moreover, laboratory tests, including Papanicolaou (Pap) tests, vaginal pH tests, and cervical biopsies were also reviewed. The participants also provided blood and cervical tissue specimens that were stored for future use. Vaginal pH was measured using pH test strips (Merck, Darmstadt, Germany) according to the manufacturer's instructions.

### Clinical laboratory tests

All participants underwent Pap smears using the LBC method. At least two cytopathologists from the Second Hospital of Shanxi Medical University evaluated the cytological results, which were then reported according to the Bethesda System 2001 terminology. Samples found to be abnormal (“atypical squamous cells of undetermined significance” or higher-stage) were further reviewed for quality control by a senior cytopathologist who was blinded to the previous pathology results. Next, gynecologic specialists from the Second Hospital of Shanxi Medical University examined abnormal cervices and obtained biopsy samples using colposcopy (SLC-2000 device, Shenzhen Goldway Industrial, Shenzhen, China) according to a standard protocol ≤12 weeks after the Pap test. Gynecologic specialists divided the cervix into quadrants and examined each. All visually abnormal areas were biopsied; quadrants without a visible lesion were biopsied at the squamocolumnar junction (“random biopsy”). The samples were classified as negative, CIN1, CIN 2, CIN3, and squamous cell carcinoma (SCC). Two pathologists performed double-blind reviews of the Pap test results when the cervical biopsy or endocervical curettage tissue specimens were found to be positive. If the pathologists had conflicting diagnoses, a third senior pathologist arbitrated. The three pathologists reviewed difficult or equivocal cases together to arrive at a consensus diagnosis.

HPV genotyping using the Hybri-Max system (Millipore-Sigma, St. Louis, MO) was performed from residual Pap test specimens using the HPV GenoArray Test Kit (Hybribio, Sheung Wan, Hong Kong), and the samples were categorized into high-risk HPV infection group vs. others (low-risk HPV type and negative). The Hybri-Max assay can identify 21 types of HPV, including the high-risk types 16, 18, 31, 33, 35, 39, 45, 51, 52, 53, 56, 58, 59, 66, and 68, as well as the low-risk types 6, 11, 42, 43, 44, and CP8304. These were detected via a flow-through hybridization technique performed with a TC-96/G/H6 HPV DNA amplification analyzer and an HMM-2 fast nucleic acid molecule hybridization instrument (Hybribio).

Vaginal pH tests were performed on the 2,304 residual Pap test specimens using pH strips (Merck). Vaginal pH values ≥4.5 were considered abnormal^29^. The pH categories were divided into two groups, normal (pH < 4.5), and abnormal (pH ≥ 4.5).

### Dietary mineral assessment

The dietary mineral intake frequencies and amounts, as determined by the FFQ, were investigated in combination with the local dietary structure of the residents of Shanxi. The FFQ was administered during an in-person interview. The FFQ in this study was nested in the standardized and structured epidemiological questionnaire of the Shanxin CIN Cohort study. The 24 h dietary recall dietary data were collected by trained interviews who recorded amounts of all the food items. Each reported frequency was assigned the average serving size for that food item^30^. The FFQ was designed based on the China Health and Nutrition Survey^8^, ^11^, ^31^, Detailed descriptions of the dietary measurements have been published elsewhere^31^,^32^. A total of 26 items food was included in the FFQ, which are main food sources for participants in this study. Based on a Chinese National Nutrition Survey in 2002^33^. The FFQ included 26 food items: wheat flour, soybeans, cabbage, egg, oat flour, bean curd, celery, cow's milk, buckwheat flour, dried bean curd, spinach, pork liver, rice, broad beans, Chinese chives, sunflower seeds, millet, potatoes, carrots, jujubes, maize, mushrooms, pumpkins, bananas, liquor, and tea. Based on the 2002 Chinese National Nutrition Survey, 10 food groups accounted for approximately 85% of the total dietary mineral intake in China^31^. The FFQ data was analyzed using the US Department of Agriculture's 1994-1996 Continuing Survey of Food Intakes^34^. Mineral intakes (grams/milligrams/micrograms per day) were then calculated by multiplying the daily food consumption amount (g per day) by the median mineral content (g per 100 g, mg per 100 mg, or μg per 100 μg) of the particular food. The mineral values from all FFQ items were combined to obtain the total daily mineral values. We estimated the level of each dietary mineral intake as the following equation: Total daily mineral value = total amount of each food (g/mg/μg) / (day)* intake value per 100 g, per 100 mg, and per 100 μg. Total mineral intake (g/mg/μg per day) was determined by calculating the sum of the daily mineral values.

### Assessing the test-retest reliability and relative validity of the FFQ

We randomly selected 218 out of 2304 people as subjects. The study started from January 2019 and lasted for the subsequent six months. During the study period, three consecutive 24-hour studies (24-HRs) were conducted every three months. The first FFQ was administered during the first 24-HR. FFQ1 was administered during the second 24-HR, and FFQ2 was administered during the last 24-HR. The 'weight estimation (WE)' method for assessing food consumed for evaluation was estimated by the respondents for the weight of each food they consumed in the previous 24 hours. The study design is shown in Supplementary Figure 1. Each participant was asked to provide the name and amount of food consumed during the previous 24 hours. If the previous day was a special day, for reasons such as banquets or travel, et al., we would record food consumption 24 hours ago, or choose another day to interview participants by telephone. Subjects were not informed of the results until the night before the interview. Record the amount of food mixed with a plate. According to the definition of food quality standard, recalled food is assigned to the corresponding food group.

Trained interviewers manage FFQ and 24 hours through face-to-face interviews. Immediately check all records and resolve any ambiguities in the subject. During the whole study period, each participant had his own interviewer. Participants who did not satisfactorily complete the FFQs or missed more than one out of the four 24-HRs were excluded from the analyses. Subjects with implausible energy intakes (<500 Kcal or >5000 Kcal) were also excluded as described by previous studies. Extreme values were examined and excluded. A decision about whether or not to exclude the record from analyses was made according to the original FFQs and/or 24-HRs. The validity of the FFQ methods was assessed by comparing the nutrient intakes derived from the FFQs. A 3-month FFQ was collected from the same subjects after administration of the first interviewer-administered FFQ.

### Statistical analysis

Descriptive statistics were used to determine the frequency, proportion, mean, and standard deviation of the demographic characteristics. Participants' characteristics were examined for significant differences using Pearson's chi-squared test for categoric variables. A logistic regression model was used to calculate the odds ratios (ORs) and their confidence intervals (CIs) for CIN risk in each nutritional ingredient quartile compared to the highest quartile. Tests of linear trend across increasing quartiles of nutritional ingredients were performed by assigning the medians of each nutritional ingredient to quartiles treated as continuous variables.

The first model was unadjusted, while subsequent analyses were adjusted for potential confounders. Multivariate models were adjusted for age (<30, 30-39, 40-49, 50-59, and ≥60 years), years of education (<6, 7-9, and >9 years), yearly income (<10,000, 10,000-30,000, and >30,000 ¥), tobacco smoking (yes, no), age at menarche (<13, 13 to <15, 15 to <17, and ≥17 years), menopausal status (yes, no), IUD use (yes, no), years of IUD use (<10 vs. ≥10 years), sexually active during the menstrual period (yes, no), history of gynecologic surgery (yes, no), and presence of vaginitis (yes, no). In the final multivariable analysis, other potential clinical confounders were added, including high-risk HPV (positive, negative), SCJ visibility (fully visualized vs. not fully visualized), vaginal pH (<4.5, ≥4.5) and dietary minerals, fitted simultaneously (iron, calcium, magnesium, phosphorus, zinc, sodium, and potassium).

Next, we performed cross-sectional analyses with three knots (25^th^, 50^th^, and 75^th^ percentiles) of the 2,304 women to examine the association between log-transformed dietary intake levels and CIN risk. This research did not explore the association between dietary intake and SCC risk because of the small number of SCC cases (n = 19). Statistical analyses were performed using SAS software version 9.3^35^. All reported *P*-values are two-sided, with a significance level of 0.05.

## Results

Table 1 shows the characteristics of the 2,304 participants. In terms of severity, 89.7% of women (n=2,076) had no advanced lesions, including had no CINs at all and had CIN1. The remaining 10.3% (n=237) had advanced lesions, including had CIN2, had CIN3, and had SCC. The mean ages (± standard deviations) of the women had no advanced lesions and had advanced lesions were 49.4 ± 9.1 and 47.3 ± 8.8 years, respectively. Women had advanced lesions were more likely to: be age 40-49 years (*P*<0.05), have experienced earlier menarche (*P*<0.05), be premenopausal (*P*<0.05), be HPV infection-positive (*P*<0.05), and use oral contraceptives (*P*<0.05). No significant differences were observed across the CIN categories in terms of education level, yearly income, smoke tobacco, IUD use, vaginal pH, vaginitis, gynecologic surgery, sexual behavior in the menstrual period, and SCJ visibility.

The median dietary mineral intake amounts in patients with no CIN as well as those with CIN1 and CIN 2+ are shown in Supplementary Table 1. Women with CIN2+ were more likely to have lower dietary mineral intakes. Table 2 shows the associations between dietary mineral intake and CIN2+ risk among the 2,304 women. After full adjustment, intake of most minerals continued to show significant associations with CIN2+ risk when using the fourth quartile as a reference. Lower intakes of dietary calcium were associated with increased risk of CIN2+. The multivariable adjusted model for CIN2+ risk revealed an OR=1.53 (95% CI: 1.02-2.31) when comparing the first quartile to the fourth (>565.9 mg per day), and an OR=0.86 (95% CI: 0.75-0.98) when comparing the total quantity to the fourth quartile (Table 2).

Comparing the group with the highest dietary magnesium intake level (>693.6 mg per day) to that with the lowest intake (<439.0 mg per day) showed significant associations with CIN2+ risk after adjustment (first quartile compared to fourth: OR=1.72 (95% CI: 1.14-2.59); total quantity compared to the fourth quartile: OR=0.82 (95% CI: 0.71-0.93) (Table 2).

An association existed between dietary phosphorus intake and risk of CIN2+ (first quartile [<1300.3 mg per day] compared to fourth [>1,998.2 mg per day] after full adjustment: OR=1.71 (95% CI: 1.13-2.59); total quantity compared to fourth quartile: OR=0.83 (95% CI: 0.73-0.95) (Table 2).

Dietary zinc intake was significantly associated with CIN2+ risk when comparing women with the lowest (<9.8 mg per day) to women with the highest (>15.2 mg per day) dietary zinc intake level, after full adjustment (first quartile compared to the fourth: OR=1.53 [95% CI: 1.02-2.30]; total quantity compared to the fourth quartile: OR=0.87 [95% CI: 0.76-0.99]) (Table 2).

A statistically significant association was also found between dietary potassium intake and risk of CIN2+ after full adjustment (first quartile compared to fourth: OR=1.90 (95% CI: 1.27-2.85); total quantity compared to the fourth quartile: OR=0.79 (95% CI: 0.70-0.91) (Table 2).

The associations between dietary mineral intake and the risk of CIN1 among the 2,304 women were identified using logistic regression analyses (Table 3). On stratified analysis, adjusting for demographics, lifestyle habits, and other covariates, we observed statistically significant associations between dietary calcium intake and CIN1 risk (second quartile compared to fourth: OR=1.36, 95% CI: 1.03-1.80), and between dietary potassium intake and CIN1 risk (second quartile compared to fourth: OR=1.90, 95% CI: 1.27-2.85). Intake of iron, magnesium, phosphorus, sodium, and zinc were not significantly associated with CIN1 risk.

The association between each dietary mineral intake and CIN risk which included CIN1 and CIN2+ (Supplementary Table 2). We observed the statistical associations between iron, calcium, magnesium, phosphorus, sodium, zinc and potassium and risk of CIN, significant associations were observed between calcium, magnesium, and potassium and CIN risk.

Dietary Potassium levels were graded by the quartile of the control group, High-risk HPV and Oral contraceptive use by the quantile of the control group. The results showed that CIN2+ risk increased as Dietary Potassium levels decreased, the Positive of High-risk HPV, and Negative of Oral contraceptive after full adjustments (Table 4).

The current study performed analyses with 25th, 50th, and 75th percentiles of the dietary Potassium in the control group was regarded. The interactions of dietary Potassium and hrHPV infection or oral contraceptive use in CIN2+ grading by stratified analysis using the addition mode were analyzed. There was a positive net interaction between low dietary Potassium and positive hrHPV infection and negative oral contraceptive in the CIN2+ groups. The controls used for OR calculation were hrHPV-positive and oral contraceptive-positive patients with highest Dietary Potassium levels, It was observed that women with hrHPV infection and lower dietary Potassium level were more likely to have CIN2+ (OR=4.22, 95% CI: 1.45-12.3); the women with negative oral contraceptive and lower dietary Potassium level were also more likely to have CIN2+ (OR=22.9, 95% CI: 2.43-217.0) than women with positive oral contraceptive and lower dietary Potassium level (OR=13.6, 95% CI: 1.21-153.5) (Table 5).

In terms of FFQ validity are shown in Supplementary Table 3, The energy-adjusted, and de-attenuated correlation coefficients of the FFQs (FFQ1 and FFQ2) and the 24-HRs are presented. The energy-adjusted correlation coefficient for WE of FFQ1; and the deattenuated coefficient, when compared with the 24-HRs. The energy-adjusted correlation coefficient for WE of FFQ2; the deattenuated coefficient, when compared with the 24-HRs, which were a little less than those of FFQ1 versus the 24-HRs. In the retest reliability of FFQ, the The intra-class correlation coefficient (ICC) of nutrient intake derived from WE FFQs collected at 3-month intervals are shown in Supplementary Table 3, All nutrients in WE-FFQ have good correlation and agreement.

## Discussion

This large-scale population-based study investigated the demographic characteristics of study subjects using an epidemiological data and a FFQ. We found that the intake levels of several dietary minerals (calcium, magnesium, phosphorus, zinc, and potassium) were significantly associated with risk of CIN2+, which may also reflect the association between dietary mineral intake and cervical carcinogenesis.

### Strength and Limitations

The main strength of the study was that it was population-based and included a large sample size for the evaluation of the association between dietary mineral intake and CIN risk, which increased its statistical power. Second, objective assessments were obtained for various factors, including squamous junction types, vaginal pH, HPV types, and CIN-related clinical examinations based on LBC, colposcopy, and cervical biopsy. These tests assured the accuracy of the laboratory test results. Third, we analyzed dose-response relationships between dietary mineral (in quartiles as well as the total quantity) and the risk of CIN1 and CIN2+; these factors were carefully adjusted using multiple potential confounders that minimized the probability of bias.

Conversely, there are several limitations of this investigation. First, causality could not be confirmed, as the study had a cross-sectional design, and we could not rule out the causality only by assigned association^34^. Since there were only 26 items on the FFQ, this limited our analysis to broad categories of foods, and the nutrient values may not have precisely reflected actual intake, given the potential for wide diversity in the actual foods consumed by individual participants for each food category. Second, misclassification of CIN cases might have occurred. Third, there may have been some residual confounding from unmeasured variables such as local lifestyle factors, that might lead to low dietary intake levels and other unhealthy behaviors that may, in turn, lead to CIN development^36^. Additionally, statistical power of small sample size CIN2+ cases is limited, which may reduce the likelihood that a statistically significant result reflects a true effect. Further studies are therefore necessary to examine an objective assessment of serum mineral levels on cervical cancer development.

### Role of multiple dietary minerals in cervical cancer

Previous epidemiologic studies supported the notion that diet and nutritional status influence cervical carcinogenesis^37^, including recent prospective studies that found that fruits and vegetables had a protective effect against cervical cancer^38^. However, the effect of dietary mineral intake was not clear, and data regarding the association between nutritional factors and the risk of cervical cancer remain sparse and inconsistent overall^39^,^40^. A previous study did reveal that potential inter-metal effects owing to the exposure to multiple minerals can affect the risk of cervical carcinogenesis^15^.

#### Calcium and phosphorus

Several epidemiological studies have shown that calcium has various anticancer effects^41^. However, a case-control study of 257 CIN patients in the state of Alabama^15^ found no significant association between dietary calcium intake and the risk of cervical cancer. More recently, East Asian case-control studies found an inverse relationship between lower dietary mineral intake and CIN2+ risk^5^,^16^, which was consistent with the current research. Such conflicting results could be due to the fact that current study based on a large-sample cohort, whereas previous investigations were case-control studies based on small population in which different potential confounders were evaluated, which may have led to varying results^42^. Moreover, inaccurate calcium content estimations in food, or substantial variations in such contents, could have contributed to the discrepancies.

Calcium has been recognized as a key component in the maintenance of proper cell structure and function, although the biological mechanisms for this remain unclear^43^. Furthermore, calcium interacts with 1,25-dihydroxyvitamin D3, which regulates cell growth, cell differentiation, and immune function in tissues^44^. However, the mechanism through which calcium may have an anti-cervical cancer effect is unclear.

Dietary phosphorus intake is reportedly associated with cancer risk^45^. A previous case-control study of 257 patients in the US found a significant association between the ratio of dietary calcium-to-phosphorus ratio and CIN risk^18^; moreover, a follow-up study of 941,471 participants in the US found that a higher dietary phosphorus intake was significantly associated with cancer risk^46^. Although it is commonly believed that a combination of low calcium and high phosphorus may be critical for the development of some cancers^47^, previous studies may have been misleading since they measured the *ratio* of dietary calcium-to-phosphorus as a CIN risk factor, rather than the dietary phosphorus intake per se. As such, dietary intake may not provide information on mineral levels in the body; while intake reflects daily normal ingestion, it does not accurately represent the levels of minerals in cells. Hence, whether phosphorus directly mediates cancer-promoting pathways is unclear^48^.

#### Magnesium and zinc

Magnesium has convincingly been shown to play a protective role in the early stages of carcinogenesis^49^. Magnesium deficiency may also be associated with increased levels of inflammatory mediators and free radicals, which in turn can cause oxidative DNA damage that contributes to tumorigenesis^50^.

Several epidemiologic studies have demonstrated that a magnesium-rich diet may reduce the occurrence of cancer^51^. A case-control study between 2002 and 2004 in Northern Ireland demonstrated the protective effect of dietary magnesium intake against the progression of precancerous lesions^52^. Moreover, a cohort study in the US similarly found that magnesium protects against esophageal squamous cell carcinoma^53^, whose histological type and mechanism of metastases are similar to those of cervical cancer^54^. These aforementioned data were consistent with the present study, which found that dietary magnesium intake is a preventative factor against cervical carcinogenesis. Future studies may focus on further elucidating the mechanisms contributing to the stages of carcinogenesis, as related to magnesium.

Zinc is an essential element that is integral to many proteins and transcription factors that regulate key cellular functions such as DNA damage repair, cell cycle progression, and apoptosis; such functions are critical for tumor suppression^55^. Zinc is also a cofactor of several enzymes involved in the synthesis of proteins and antioxidants^56^. A previous epidemiologic case-control study in Oregon found that zinc was not protective against risk of high-grade squamous intraepithelial lesions^56^. Conversely, a cross-sectional study in Mexico found that dietary zinc was protective against squamous intraepithelial lesions^19^, which was consistent with the results of the current research. These conflicting results may be due to the preliminary studies having small sample sizes and to the effect of dietary micronutrient intake measurement errors. Using an FFQ (as was done in the previous study) meant that the participants were not asked about some possible contributors to dietary zinc; as such, dietary consumption of some foods with the high zinc content could not be assessed. Therefore, the effect of zinc in the diet requires closer investigation in future studies.

#### Potassium and iron

Recent case-control studies found that a high intake of dietary potassium reduces the risk of cancer^23^, ^57^. However, a case-control study and another cohort study found that dietary potassium intake was not associated with cancer risk^58^,^59^. Although few studies found an association between dietary potassium and cervical cancer, current research suggest that dietary potassium could be carcinogenic at certain doses that nutritionists should be aware of. Potassium was found to have a protective effect against CIN2+ at certain doses below the mean total intake; the underlying mechanism of the relationship between potassium and CIN requires further investigation.

A previous study found that increased dietary iron as well as serum ferritin had anti-CIN effects^21^, whereas this study found that dietary iron was not significantly associated with the risk of CIN2+. These conflicting results may be due to the FFQ not accurately representing iron levels, potentially due to recall bias, or information bias. Whether dietary iron is significantly associated with risk of cervical cancer should be investigated in larger cohorts.

### High-risk HPV infection and oral contraceptives

It is well established that infection with high-risk HPV types is a necessary cause for cervical cancer[60. Consistent with prior research, we observed that high-risk HPV infection was associated with increased prevalence of CIN compared with no high-risk HPV infection among women in this study. We also observed evidence for a interaction between lower dietary Potassium level and no oral contraceptives and for the risk of CIN2+, which may partly account for the high incidence of cervical cancer in our cohort that Potassium intake deficiency and high prevalence of no oral contraceptives among Chinese women in Shanxi province. So far, no information exploring interactions between dietary Potassium and oral contraceptives with cervical cancer is available from epidemiologic studies. This makes sense as diet consumption is associated with immunity through a variety of mechanisms^61^ (intestinal malabsorption, reduced liver uptake and storage, urinary excretion, Intestinal Microecology, etc).

## Conclusion

Dietary intake of calcium, magnesium, phosphorus, zinc, and potassium was associated with risk of CIN2+. Synergy may potentially exist between low dietary potassium and hrHPV infection or negative oral contraceptive to promote CIN development. These findings support the result that lower minerals intake was an independent risk factor contribute to development of higher-grade CIN and cervical cancer.

## Supplementary Material

Supplementary figure and tables.

## Figures and Tables

**Table 1:** Basic Characteristics with cervical histological examination among 2,304 women with atypical squamous cells of undetermined significance and above in a cohort study

Characteristics^ a^	Total	had no advanced lesions^b^	had advanced lesions^c^	*P* value^d^
No. of participants (%)	2,304 (100.0)	2067 (89.7)	237 (10.3)	
Age (years)	49.2 ± 9.1	49.4 ± 9.1	47.3 ± 8.8	
< 30	58 (2.5)	55 (2.7)	3 (1.3)	
30-39	284 (12.3)	243 (11.8)	41 (17.3)	*P*= 0.04^*^
40-49	711 (30.9)	616 (29.8)	95 (40.1)	
50-59	970 (42.1)	897 (43.4)	73 (30.8)	
> 60	281 (12.2)	256 (12.4)	25 (10.5)	
Educational years				
0-6	472 (20.5)	426 (20.6)	46 (19.4)	
7-9	996 (43.2)	869 (42.0)	127 (53.6)	*P= 0.05*
> 9	836 (36.3)	772 (37.3)	64 (27.0)	
Yearly Income^e^ (¥)				
< 10,000	262 (11.4)	238 (11.5)	24 (10.1)	
10,000-30,000	1119 (48.6)	993 (48.0)	126 (53.2)	*P= 0.76*
> 30,000	923 (40.1)	836 (40.4)	87 (36.7)	
Tobacco smoking	49 (2.1)	41 (2.0)	8 (3.4)	*P= 0.06*
Menopause	1174 (51.0)	1096 (53.0)	78 (32.9)	P< 0.01*
High-risk HPV^f^				
Positive	755 (32.8)	597 (29.0)	158 (66.7)	P< 0.01*
Negative	1549 (67.2)	1470 (71.1)	79 (33.3)	
Menarche age	15.0 ± 2.1	15.1 ± 2.2	14.6 ± 1.9	
<13	308 (13.4)	274 (13.3)	34 (14.4)	
13-14	739 (32.1)	646 (31.3)	93 (39.2)	
15-16	654 (28.4)	585 (28.3)	69 (29.1)	P= 0.03*
>17	603 (26.2)	562 (27.2)	41 (17.3)	
Intrauterine device use	1081 (46.9)	966 (46.7)	115 (48.5)	*P= 0.89*
Intrauterine use years				
<10	1632 (70.8)	1463 (70.8)	169 (71.3)	*P= 0.29*
≥10	672 (29.2)	604 (29.2)	68 (28.7)	
SCJ^g^ **visibility**				
fully visible	646 (28.0)	567 (27.4)	79 (33.3)	*P= 0.11*
not fully visible	1658 (72.0)	1500 (72.6)	158 (66.7)	
Vaginal pH				
<4.5	514 (22.3)	447 (21.6)	67 (28.3)	*P= 0.23*
≥4.5	1790 (77.7)	1620 (78.4)	170 (71.7)	
Gynecologic surgery	397 (17.2)	362 (17.5)	35 (14.8)	*P= 0.65*
Vaginitis	140 (6.1)	120 (5.8)	20 (8.4)	*P= 0.11*
Bathing after intercourse	1832 (79.5)	1634 (79.1)	198 (83.5)	*P= 0.06*
Oral contraceptive use	172 (7.5)	162 (7.8)	10 (4.2)	P< 0.01*
Sexual behavior activity during the menstrual period	56 (2.4)	48 (2.3)	8 (3.4)	*P= 0.55*

^a^: Data were presented as number (%) of participants. ^b^: Had no advanced lesions included without CIN and CIN1. ^c^: Had advanced lesions included CIN 2, CIN 3 and SCC. ^d^: *P* values for differences between groups were obtained from the chi-square test for categorical categoric variables. ^e^: Represent in terms of Chinese Renminbi (RMB). ^f^: HPV= human papilloma virus. ^g^: SCJ=Squamous-columnar junction. ^*^Significant estimates (P<0.05).

**Table 2:**
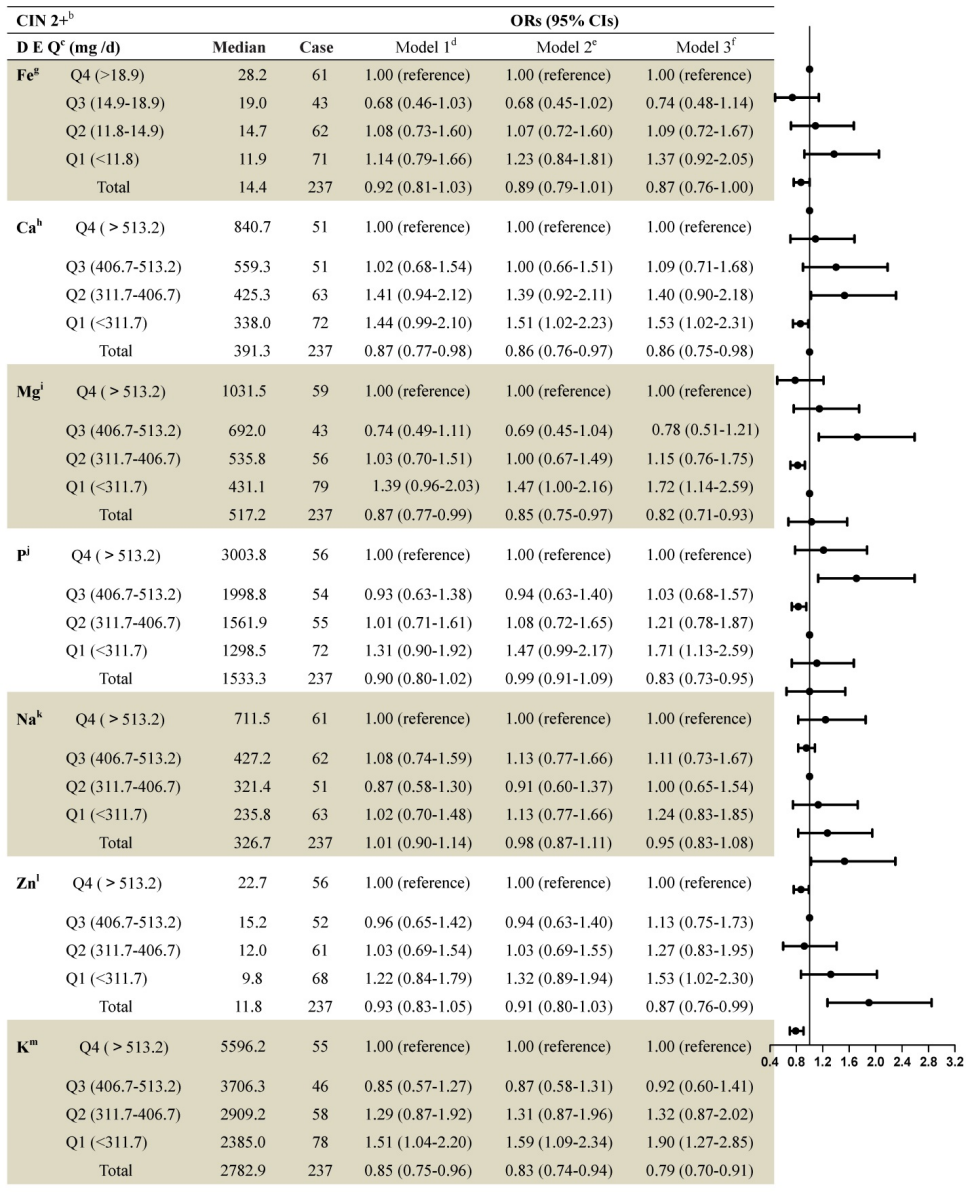
ORs and 95% Cls for quintiles of dietary Element intake with cervical intraepithelial neoplasia (grade 2 and above) risk among 2,304 women in the study^a^

^a^: Values are n or ORs (95% CIs) obtained from logistic regression analysis, based on the highest intake group as the reference, unless otherwise indicated. ^b^: CIN2+ included CIN 2, CIN 3 and SCC. ^c^: DEQ= dietary element quintiles. ^d^: Model 1 OR: unadjusted; ^e^: Model 2 OR: adjusted for education; income; smoke; menarche age; menopause; ^f^: Model 3 OR: additionally odds ratios adjusted for age; HPV; the sex life cleans; intrauterine device use; intrauterine use year; SCJ visibility; vaginal pH; menstrual sexual behavior; gynecologic surgery; vaginitis; Bathe after sexual behavior; Oral contraceptive use. ^g^: Fe= Iron; ^h^:Ca= Calcium; ^I^:Mg= Magnesium; ^ j^:P= Phosphorus; ^k^:Na= Sodium; ^l^:Zn= Zinc; ^m^:K= Potassium.

**Table 3:**
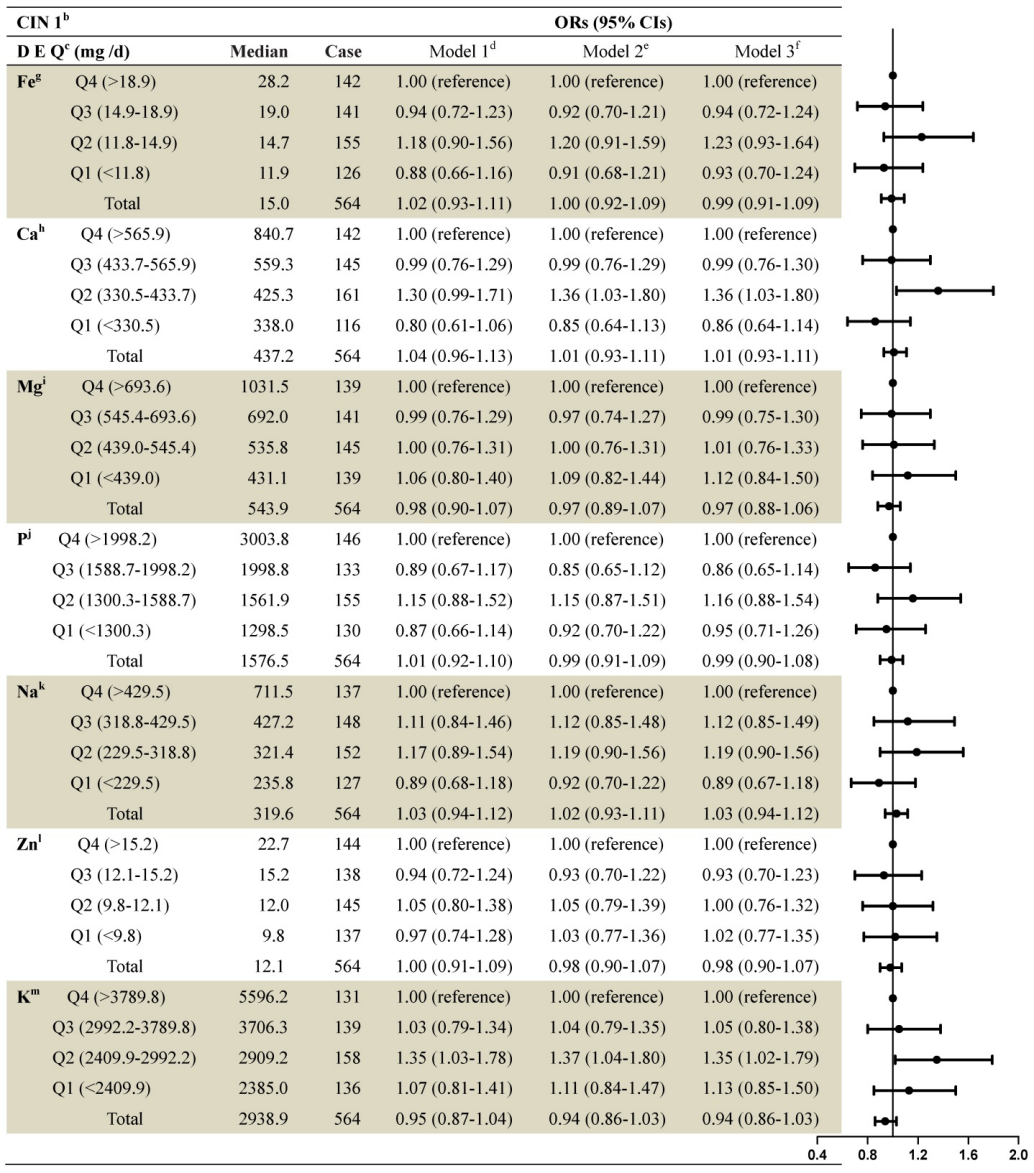
ORs and 95% Cls for quintiles of dietary element intake with cervical intraepithelial neoplasia (grade 1) risk among 2,304 women in the study^a^

^a^: Values are n or ORs (95% CIs) obtained from logistic regression analysis, based on the highest intake group as the reference, unless otherwise indicated. ^b^: CIN1= cervical intraepithelial neoplasia grade 1. ^c^: DEQ= dietary element quintiles. ^d^: Model 1 OR: unadjusted; ^e^: Model 2 OR: adjusted for education; income; smoke; menarche age; menopause; ^f^: Model 3 OR: additionally odds ratios adjusted for age; HPV; the sex life cleans; intrauterine device use; intrauterine use year; SCJ visibility; vaginal pH; menstrual sexual behavior; gynecologic surgery; vaginitis; Bathe after sexual behavior; Oral contraceptive use. ^g^: Fe= Iron; ^h^:Ca= Calcium; ^I^:Mg= Magnesium; ^ j^:P= Phosphorus; ^k^:Na= Sodium; ^l^:Zn= Zinc; ^m^:K= Potassium.

**Table 4:** ORs and 95% CIs for the associations between Dietary Potassium, high-risk HPV infection and Oral contraceptive use with the risk of cervical intraepithelial neoplasia grades 2 and above^a^

	Participants, n		ORs (95% CIs)^b^		
	Normal	Case	Model 1	Model 2	Model 3
CIN2+					
Dietary Potassium, (mg /d)					
Q4 (>513.2)	390	55	1.00 (reference)	1.00 (reference)	1.00 (reference)
Q3 (406.7-513.2)	391	46	0.83 (0.55-1.27)	1.08 (0.59-1.98)	1.08 (0.57-2.05)
Q2 (311.7-406.7)	360	58	1.14 (0.77-1.70)	1.65 (0.75-3.63)	1.59 (0.68-3.69)
Q1 (<311.7)	362	78	1.53 (1.05-2.22)	2.71 (1.08-6.81)	3.31 (1.21-9.03)
High-risk HPV					
Positive	429	158	1.00 (reference)	1.00 (reference)	1.00 (reference)
Negative	1074	79	0.20 (0.15-0.27)	0.19 (0.14-0.26)	0.21 (0.15-0.29)
Oral contraceptive use					
Positive	113	10	1.00 (reference)	1.00 (reference)	1.00 (reference)
Negative	1390	227	1.85 (0.95-3.58)	1.93 (0.99-3.77)	2.74 (1.34-5.59)

^a^:Values are n or ORs (95% CIs) obtained from logistic regression analysis, using the highest intake group as the reference, unless otherwise indicated. CIN2+ = cervical intraepithelial neoplasia grade 2 and above. ^b^:Model 1: unadjusted. Model 2: adjusted for Dietary Iron; Calcium; Magnesium; Phosphorus; Sodium; Zinc; Potassium. Model 3: odds ratios adjusted for age education; income; smoke; menarche age; menopause; HPV; the sex life cleans; intrauterine device use; intrauterine use year; SCJ visibility; vaginal pH; menstrual sexual behavior; gynecologic surgery; vaginitis; Bathe after sexual behavior; Oral contraceptive use.

**Table 5:** ORs and 95% CIs for cervical intraepithelial neoplasia grades 2 and above with the risk of the Dietary Potassium in the hrHPV infection, Oral contraceptive use and control group^a^

CIN2+			ORs (95% CIs)^b^			ORs (95% CIs)^b^	
Dietary Potassium		hrHPV			OC use		
	N		Model 1	Model 2		Model 1	Model 2
Q4 (>513.2)	55	+	Reference	Reference	+	Reference	Reference
Q4 (>513.2)		-	0.26 (0.14-0.46)	0.27 (0.15-0.50)	-	4.99 (0.67-37.2)	7.36 (0.95-57.4)
Q3 (406.7-513.2)	46	+	0.84 (0.48-1.48)	1.03 (0.49-2.16)	+	2.28 (0.20-26.4)	4.11 (0.32-53.4)
Q3 (406.7-513.2)		-	0.24 (0.13-0.44)	0.31 (0.14-0.67)	-	4.01 (0.54-30.0)	7.52 (0.92-61.7)
Q2 (311.7-406.7)	58	+	1.54 (0.92-2.59)	1.96 (0.78-4.90)	+	1.44 (0.09-24.1)	1.95 (0.10-38.9)
Q2 (311.7-406.7)		-	0.21 (0.11-0.40)	0.32 (0.12-0.85)	-	5.58 (0.75-41.6)	11.5 (1.30-101.1)
Q1 (<311.7)	78	+	2.01 (1.22-3.32)	4.22 (1.45-12.3)	+	7.07 (0.80-62.3)	13.6 (1.21-153.5)
Q1 (<311.7)		-	0.33 (0.19-0.57)	0.69 (0.23-2.02)	-	7.11 (0.96-52.8)	22.9 (2.43-217.0)

^a^: Values are n or ORs (95% CIs) obtained from logistic regression analysis, using the highest intake group as the reference, unless otherwise indicated. CIN2+ = cervical intraepithelial neoplasia grade 2 and above; hrHPV = High-risk human papilloma virus; OC use= Oral contraceptive use. ^b^: Model 1: unadjusted. Model 2: adjusted for Dietary Iron; Calcium; Magnesium; Phosphorus; Sodium; Zinc; Potassium, and additionally adjusted for age education; income; smoke; menarche age; menopause; HPV; the sex life cleans; intrauterine device use; intrauterine use year; SCJ visibility; vaginal pH; menstrual sexual behavior; gynecologic surgery; vaginitis; Bathe after sexual behavior; Oral contraceptive use.

## Abbreviations

CIN: cervical intraepithelial neoplasia
HPV: human papilloma virus
IUD: intrauterine device
SCJ: squamous-columnar junction
OR: odds ratio
CI: confidence interval
