# Supplementary material for: Lower dietary mineral intake is significantly associated with cervical cancer risk in a population-based cross-sectional study
Source: J Cancer. 2021 Jan 1;12(1):111–23. doi: 10.7150/jca.39806 (PMC7738836; doi:10.7150/jca.39806)
Supplement: Supplementary file 1 — Supplementary figure and tables. [file jcav12p0111s1.pdf]

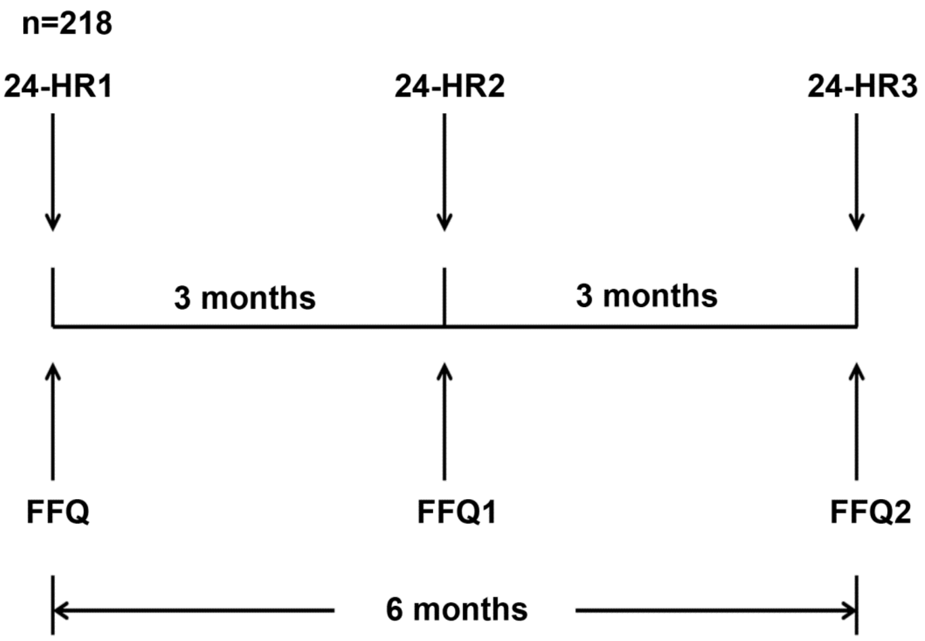

Supplementary Figure 1 Design of the reproducibility and validation study. FFQ was administered during the first 24-HR, FFQ1 was administered during the second 24-HR, and FFQ2 was administered during the last 24-HR. The three 24-HRs were administered at intervals of three months.

Supplementary Table 1. Dietary nutrition intake were investigated based on FFQ[a] among the 2,304 ASC-US+ Chinese women in the Shanxi Cohort Study[b]

**Element** (mg/day)	**Without CIN**	**CIN 1**	**CIN 2+**	**Total**
**Iron**	15.0 (1.4-97.7)	15.0 (2.4-90.4)	14.4 (3.5-47.2)	14.9
**Calcium**	437.5 (71.6-2011.0)	437.2 (62.3-3000.0)	391.3 (79.7-1461.5)	433.7
**Magnesium**	550.9 (50.0-3718.8)	543.9 (102.5-3587.5)	517.2 (120.7-3138.3)	545.4
**Phosphorus**	1599.6 (157.6-9807.5)	1576.5 (276.7-9217.5)	1533.3 (487.7-8126.2)	1588.7
**Sodium**	318.1 (21.8-1572.2)	319.6 (19.3-2054.2)	326.7 (17.5-969.8)	318.8
**Zinc**	12.1 (1.1-70.8)	12.1 (1.9-74.4)	11.8 (2.9-57.2)	12.1
**Potassium**	3032.1 (428.2-11974.7)	2938.9 (527.5-19535.8)	2782.9 (812.8-8911.2)	2992.2

[a] : FFQ= food frequency questionnaires.

[b] : Data were presented as Median with range.

Supplementary Table 2. ORs and 95% Cls for quintiles of dietary Element intake with cervical intraepithelial neoplasia risk among 2,304 women in the study[a]

CIN[b]				ORs (95% CIs)		
**D E Q[c] (mg /d)**		**Median**	**Case**	Model 1[d]	Model 2[e]	Model 3[f]
**Fe[g]**	Q4 (>18.9)	28.2	142	1.00 (reference)	1.00 (reference)	1.00 (reference)
	Q3 (14.9-18.9)	19.0	141	0.85 (0.67-1.08)	0.84 (0.66-1.07)	0.87 (0.68-1.11)
	Q2 (11.8-14.9)	14.7	155	1.11 (0.88-1.41)	1.14 (0.90-1.45)	1.16 (0.91-1.47)
	Q1 (<11.8)	11.9	126	0.98 (0.77-1.25)	1.03 (0.81-1.31)	1.06 (0.83-1.35)
	Total	15.0	564	1.00 (0.99-1.01)	1.00 (1.00-1.01)	1.00 (1.00-1.00)
**Ca[h]**	Q4 (>565.9)	840.7	142	1.00 (reference)	1.00 (reference)	1.00 (reference)
	Q3 (433.7-565.9)	559.3	145	1.02 (0.80-1.30)	1.02 (0.80-1.30)	1.06 (0.83-1.35)
	Q2 (330.5-433.7)	425.3	161	1.26 (0.99-1.59)	1.30 (1.02-1.64)	1.31 (1.03-1.67)
	Q1 (<330.5)	338.0	116	1.02 (0.80-1.06)	1.05 (0.82-1.34)	1.06 (0.83-1.36)
	Total	437.2	564	1.00 (1.00-1.00)	1.00 (1.00-1.00)	1.00 (1.00-1.00)
**Mg[i]**	Q4 (>693.6)	1031.5	139	1.00 (reference)	1.00 (reference)	1.00 (reference)
	Q3 (545.4-693.6)	692.0	141	0.87 (0.69-1.11)	0.85 (0.67-1.09)	0.90 (0.70-1.15)
	Q2 (439.0-545.4)	535.8	145	1.01 (0.80-1.28)	1.02 (0.80-1.29)	1.03 (0.81-1.32)
	Q1 (<439.0)	431.1	139	1.20 (0.95-1.52)	1.24 (0.98-1.57)	1.31 (1.03-1.66)
	Total	543.9	564	1.00 (1.00-1.00)	1.00 (1.00-1.00)	1.00 (1.00-1.00)
**P[j]**	Q4 (>1998.2)	3003.8	146	1.00 (reference)	1.00 (reference)	1.00 (reference)
	Q3 (1588.7-1998.2)	1998.8	133	0.90 (0.71-1.14)	0.90 (0.71-1.15)	0.94 (0.74-1.21)
	Q2 (1300.3-1588.7)	1561.9	155	1.05 (0.83-1.33)	1.15 (0.87-1.51)	1.13 (0.89-1.44)
	Q1 (<1300.3)	1298.5	130	1.04 (0.82-1.32)	1.09 (0.86-1.38)	1.15 (0.90-1.47)
	Total	1576.5	564	1.00 (1.00-1.00)	1.00 (1.00-1.00)	1.00 (1.00-1.00)
**Na[k]**	Q4 (>429.5)	711.5	137	1.00 (reference)	1.00 (reference)	1.00 (reference)
	Q3 (318.8-429.5)	427.2	148	1.08 (0.86-1.37)	1.11 (0.87-1.40)	1.13 (0.88-1.43)
	Q2 (229.5-318.8)	321.4	152	1.01 (0.79-1.28)	1.04 (0.82-1.32)	1.07 (0.84-1.36)
	Q1 (<229.5)	235.8	127	0.95 (0.75-1.21)	1.00 (0.78-1.27)	1.01 (0.79-1.30)
	Total	319.6	564	1.00 (1.00-1.00)	1.00 (1.00-1.00)	1.00 (1.00-1.00)
**Zn[l]**	Q4 (>15.2)	22.7	144	1.00 (reference)	1.00 (reference)	1.00 (reference)
	Q3 (12.1-15.2)	15.2	138	0.95 (0.74-1.21)	0.94 (0.73-1.20)	0.98 (0.76-1.26)
	Q2 (9.8-12.1)	12.0	145	1.04 (0.81-1.32)	1.04 (0.82-1.33)	1.07 (0.84-1.37)
	Q1 (<9.8)	9.8	137	1.05 (0.82-1.33)	1.09 (0.85-1.39)	1.14 (0.89-1.46)
	Total	12.1	564	1.00 (0.99-1.02)	1.00 (0.98-1.01)	1.00 (0.98-1.01)
**K[m]**	Q4 (>3789.8)	5596.2	131	1.00 (reference)	1.00 (reference)	1.00 (reference)
	Q3 (2992.2-3789.8)	3706.3	139	0.97 (0.76-1.24)	0.97 (0.76-1.25)	1.01 (0.79-1.30)
	Q2 (2409.9-2992.2)	2909.2	158	1.23 (0.97-1.56)	1.26 (0.99-1.60)	1.27 (0.99-1.62)
	Q1 (<2409.9)	2385.0	136	1.28 (1.01-1.62)	1.33 (1.04-1.69)	1.38 (1.08-1.76)
	Total	2938.9	564	1.00 (1.00-1.00)	1.00 (1.00-1.00)	1.00 (1.00-1.00)

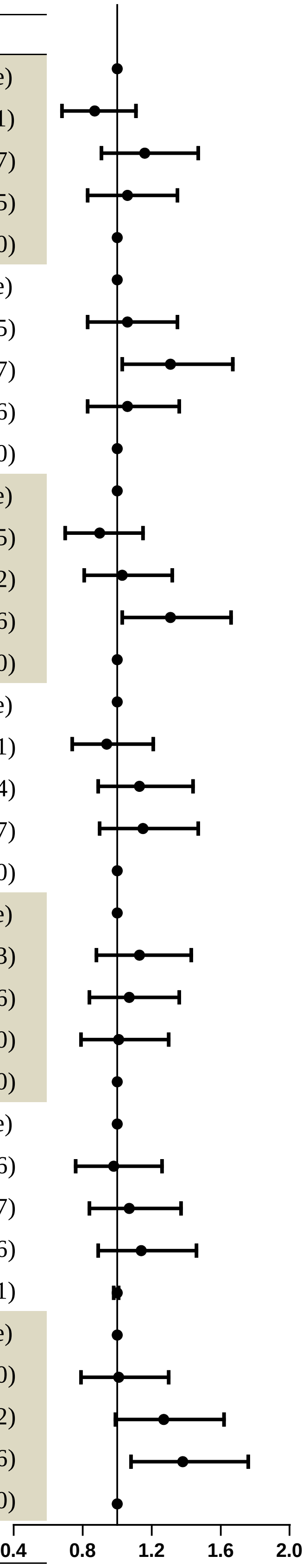

[a]: Values are n or ORs (95% CIs) obtained from logistic regression analysis, based on the highest intake group as the reference, unless otherwise indicated.

[b]: CIN included CIN, CIN 2, CIN 3 and SCC.

[c]: DEQ= dietary element quintiles.

[d]: Model 1 OR: unadjusted;

[e]: Model 2 OR: adjusted for education; income; smoke; menarche age; menopause;

[f]: Model 3 OR: additionally odds ratios adjusted for age; HPV; the sex life cleans; intrauterine device use; intrauterine use year; SCJ visibility; vaginal pH; menstrual sexual behavior; gynecologic surgery; vaginitis; Bathe after sexual behavior; Oral contraceptive use.

[g]: Fe= Iron; [h]:Ca= Calcium; [I]:Mg= Magnesium; [j]:P= Phosphorus; [k]:Na= Sodium; [l]:Zn= Zinc; [m]:K= Potassium.

Supplementary Table 3. Reliability and validity of food-frequency questionnaires[a] (24-HRs, FFQ1 and FFQ2) in 218 women in China.

**Variables**	**FFQ1 vs. 24-HRs**		**FFQ2 vs. 24-HRs**		**FFQ1 vs. FFQ2**	
Dietary elements	r-Energy adjusted[b]	r-Deattenuated[c]	r-Energy adjusted[b]	r-Deattenuated[c]	ICC[d]	*P* value[e]
Vitamin B1 (mg)	0.91	0.91	0.95	0.93	0.89	<0.001*
Vitamin B6 (mg)	0.96	0.95	0.97	0.97	0.90	<0.001*
Vitamin C (mg)	0.95	0.89	0.90	0.83	0.92	<0.001*
Vitamin K (μg)	0.99	0.78	0.98	0.79	0.94	<0.001*
Energy (Kcal)					0.90	<0.001*

[a] Data were log-transformed.

[b] Data were log-transformed and energy-adjusted correlation coefficients.

[c] Data were log-transformed and de-attenuated correlation coefficients.

[d] Data were log-transformed and intra-class correlation coefficients.

[e] *P* value of intraclass correlation coefficients between two FFQ administrations. *Significant estimates (P<0.05).
